# A Review: Using Multiple Templates for Molecular Imprinted Polymer: Is It Good?

**DOI:** 10.3390/polym14204441

**Published:** 2022-10-20

**Authors:** Niky Murdaya, Anastasya Leatemia Triadenda, Driyanti Rahayu, Aliya Nur Hasanah

**Affiliations:** 1 Department of Pharmaceutical Analysis and Medicinal Chemistry, Faculty of Pharmacy, Padjadjaran University, Bandung 45363, Indonesia; 2 Drug Development Study Center, Faculty of Pharmacy, Universitas Padjadjaran, Bandung 45363, Indonesia

**Keywords:** molecularly imprinted polymer, multi-template, drugs, organic molecules, proteins

## Abstract

A multi-template molecularly imprinting polymer (MT-MIP) strategy has been proposed and is increasingly utilised to synthesise MIP with multiple recognition sites in a single polymer using multiple target species as templates. This approach can expand MIP applications for simultaneous recognition and extraction of more than one analyte. The advantages of MT-MIP are simultaneous analyte extraction in one process, lower solvent consumption, cost-effectiveness, and short analysis time. The use of multiple templates to prepare a MIP reduces the effort required to prepare different MIPs for different analytes separately. Although there are many studies about developing MT-MIP, there are no review articles that discuss the success rate of MT-MIP. Therefore, in this review, we summarise MT-MIP synthesis, including the polymerisation method being used, the important factors that affect the quality of MT-MIP, and MT-MIP applications. MT-MIP has great potential in chemical isolation and analysis. MT-MIP produces a product that has good sensitivity, selectivity, and reusability. Furthermore, many templates, functional monomers, and crosslinkers can be formulated as MT-MIP and have a high success rate. This is evidenced by the good values of the maximum absorption capacity (Qmax), imprinting factor (IF), and reusability. We expect that the evidence presented in this review can encourage additional research on the development and application of MT-MIP.

## 1. Introduction

Molecularly imprinted polymer (MIP) has selective binding sites in the form of cavities or pores formed due to template removal through an extraction process after polymer formation. The function of the selective binding sites is to identify target molecules with the same sizes, shapes, structures, and properties as the template [1,2]. MIPs have numerous advantages, including high selectivity, affinity, and physical and chemical stability, ease of preparation, low costs, and resistance to harsh environmental conditions [3]. As a result, molecular imprinting has received a lot of attention and is now widely used for a variety of target molecules, including chemicals and drugs.

**Figure 1:**
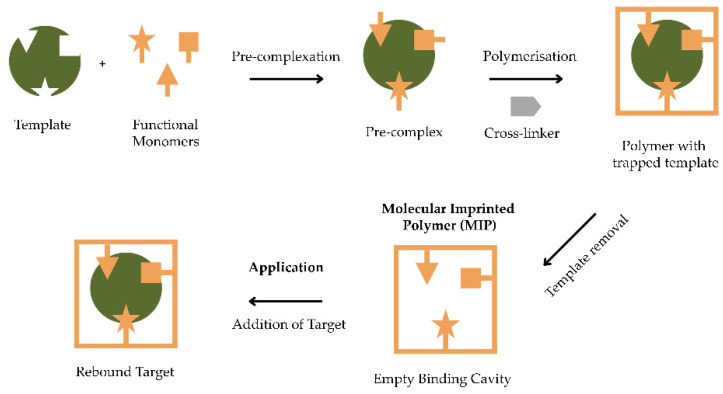
Illustration of molecularly imprinted template production.

MIP components comprise a template, a functional monomer, a crosslinker, a solvent (called porogen), and an initiator. These components have an important role in the process of forming the MIP [4]. Molecular detection in MIPs depends on the interaction between the template and the functional monomer by the formation of a suitable cavity. The functional monomers must be able to provide suitable functional groups to form a stable complex with the template [5]. The monomers commonly used are methacrylic acid (MAA), acrylamide (AM), 4-vinylpyridine (4-VP), and itaconic acid [6]. In the process of preparing a MIP, the crosslinker fixes the functional monomer bonds around the template so that the rigid structure of the MIP remains unchanged after template removal. In other words, the crosslinker functions as an adhesive/glue to secure the polymer′s shape and maintain its stability [7]. Commonly used crosslinkers are dicumyl peroxide, triallyl isocyanurate, *N*,*N*-methylendiacylamide, ethylene glycol dimethacrylate (EGDMA), tetraethoxysilane, and diphenyldiethoxysilane [6]. The solvent used is generally called a porogen (pore generator) and also acts as a dispersion agent [4]. Commonly used solvents are chloroform, acetonitrile, *N*,*N*-dimethylformamide (DMF), dichloroethane, methanol, tetrahydrofuran (THF), 2-methoxyethanol, and toluene [6]. The formation of MIPs by the free radical polymerisation method requires a free radical source. In this case, an initiator is used as a source of free radicals [4]. Commonly used initiators are azobis (nitriles) and peroxides [6]. Figure 1 provides an illustration of how MIPs are synthesised.

In the vast majority of cases, the molecule chosen as the template is the one that must be extracted by the MIP, ensuring optimal recognition during the extraction process [5]. In addition, there are other factors to consider when choosing a template, such as selecting a template with a low cost, a large molecular volume so that it is not easy to enter or exit the MIP′s moulding cavity, and thermal stability [8]. 

A single template molecule is commonly used to create a MIP that is highly selective for a target analyte [9,10]. Table 1 provides a summary of templates that have been used to generate single-template MIPs (ST-MIPs). Although ST-MIP has good selectivity and sensitivity, it is less effective when applied to the isolation or analysis of more than one compound. This is because new MIP synthesis is required for each different compound. However, for practical purposes, imprinting procedures are not limited to a single template [11].

To synthesise MIPs with multiple types of recognition sites in one format using dual/multiple targets/species as templates, a multi-template imprinting strategy has been proposed and is being used more frequently. This strategy can expand the applications of MIPs for simultaneous recognition and extraction of multiple analytes [9]. The use of multi-template MIPs (MT-MIPs) has been developed because they have several advantages over ST-MIPs, including simultaneous analyte extraction in one process, lower solvent consumption, cost effectiveness, and short analysis time. The use of multiple MIP templates reduces the effort required to prepare different MIPs for different analytes separately [26].

We believe that preparing MT-MIPs is necessary, and these products have very good future prospects. This is evidenced by the abovementioned advantages of MT-MIPs. Although there are many studies about developing MT-MIPs, there are no review articles that discuss the success rate of MT-MIPs. Therefore, in this review, we summarise the MT-MIP synthesis process, including the polymerisation method being used, the important factors that affect the quality of MT-MIPs, and MT-MIPs applications. In addition, we present the advantages of MT-MIPs over ST-MIPs and the success rate of using various types of templates and functional monomers in the synthesis of MT-MIPs. We expect that this review will encourage additional research on the discovery and development of MT-MIPs.

## 2. Multi-Template Strategy

### 2.1. Multi-Template Structure

**Figure 2:**
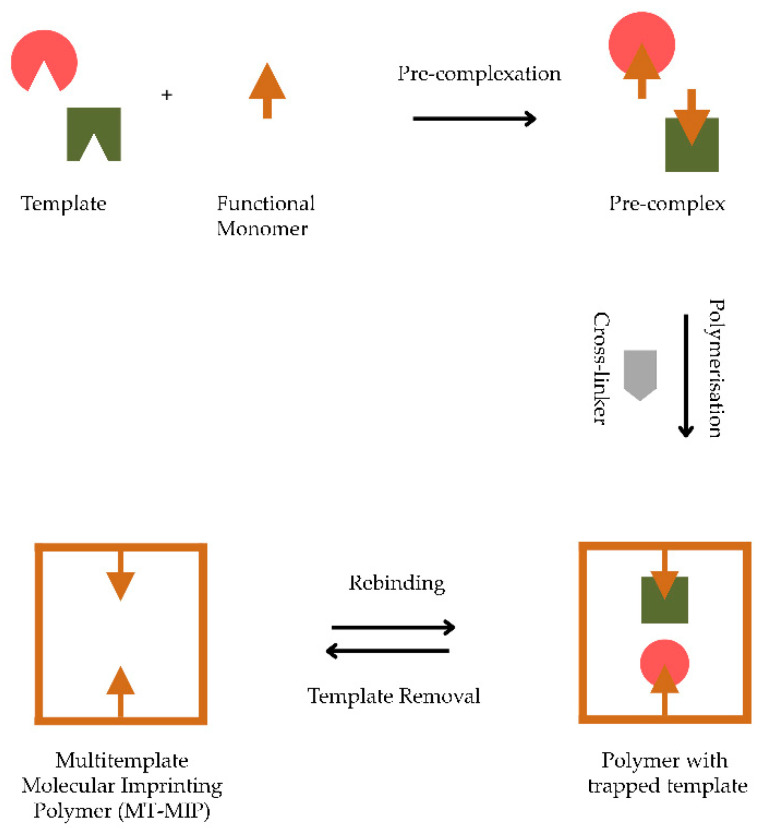
Illustration of the multi-template molecularly imprinted polymer production.

Figure 2 shows a schematic of MT-MIP synthesis. It begins with dissolving the templates, functional monomer, crosslinker, and initiator in a solvent (porogen) [27]. The template interacts with the functional monomers to form a complex. This complex is called prepolymerisation because the polymer structure has formed but is not yet rigid [28]. Therefore, a crosslinker is added, which surrounds the complex and makes the complex structure rigid and stable [29]. The next stage is the template removal process. This step is usually conducted by extraction using a solvent. After the template removal process, the expected MT-MIP is obtained [30,31].

### 2.2. Polymerisation Method

The polymerisation method for an MT-MIPs is the same as for an ST-MIPs. What distinguishes the two methods is the composition of each template, functional monomer, crosslinker, and solvent used. An MT-MIPs uses two or more templates, and this approach affects the selection of the other reagents [32,33]. Table 2 presents a summary of the polymerisation methods for MT-MIPs with the advantages and disadvantages of each method.

The most common and widely used polymerisation method in the manufacture of ST-MIPs and MT-MIPs is bulk polymerisation (see Tables 1, 3–6). This is because bulk polymerisation is universal, simple, and easy [35]. This method requires sieving and grinding after polymer formation because the polymer is formed in bulk [58]. This sieving and grinding tend to produce various shapes and sizes of particles. In addition, this process damages the binding cavity, thereby reducing the effectiveness of polymerisation [37]. In MT-MIP synthesis (see Tables 3–6), bulk polymerisation is compatible with most monomers, namely MAA, AM, 3-aminopropyltriethoxysilane (APTES), 2-vinylpyridine (2-VP), and 4-VP. Bulk polymerisation can be used for templates such as drugs, organic compounds, and proteins. Jafari et al. [59] reported very good absorption capacity and imprinting factor (IF) values. This shows that MT-MIP has good quality even though it has gone through sieving and grinding [59].

Precipitation polymerisation is also widely used for MT-MIP synthesis because it is a one-step preparation, produces uniform shapes and sizes, and has a high yield [60]. This polymerisation occurs in a solution/solvent; then, the polymer particles form precipitate from the solution. Therefore, this method requires a lot of solvents and templates [40]. Chauhan et al. [61] used precipitation polymerisation with MAA as the functional monomer and produced an average particle size of 300 nm. Abdulhussein et al. [62] also synthesised MT-MIP using precipitation polymerisation. They used 2-VP and obtained particles >50 nm. This variation in particle size occurs because of the stirring factor and the nature of the template [40,63].

Multi-step swelling/seed polymerisation is another method that can be used for the synthesis of MT-MIP. The polymerisation process with this method is quite difficult and complicated because it requires specific reaction conditions [64]. In this method, the initiator′s oil-in-water emulsion is used to generate spherical particles, which then swell [53]. The method produces particles that are monodisperse in size and shape. In addition to the controlled size, the particles formed are directly in the form of beads [55]. Luo et al. [65] synthesised MT-MIP using the multi-step swelling/seed polymerisation method with 2-VP and produced monodisperse particles with a size of 3–5 μm (the expected size).

We also found several studies that synthesised MT-MIPs using solvothermal polymerisation (see Table 6). This process occurs in a solvent at a temperature greater than the solvent′s boiling temperature [66,67]. Gao et al. [67] synthesised MT-MIPs with APTES and phenyltrimethoxysilane (PTMOS) and obtained a particle size of 100 nm. This size was obtained due to the temperature and stirring factors.

### 2.3. Influencing Factors

#### 2.3.1. Template

Templates are a crucial component in the MIP production process. This is because templates affect the selection of functional monomers, crosslinkers, initiators, and porogens [28]. The templates themselves are usually the target compounds that will be used for analysis [68]. The templates used in MIP synthesis must be able to form stable complexes with functional monomers. To form stable complexes, templates must have inert functional groups. In addition, templates must have good temperature and chemical stability [69] so that they do not degrade during polymerisation. Some polymerisation processes require heat, radical compounds, and porogens, which can affect the stability of the template [70,71]. In making MT-MIP, these conditions also apply.

Michael et al. [72] stated that the molecular weight that produces the best MIP is in the range of 200–1200 Da. In general, templates can be divided into four groups, namely ions (Cu^2+^, Cd^2+^, and Pb^2+^), organic molecules (drugs and chemicals), biomacromolecules (albumin and adenosine), and cells (viruses) [27]. So far, the templates used in MT-MIP have mostly been compounds in the same group [32,33,73,74,75]. The selection of compounds in the same group aims to facilitate the easy selection of functional monomers, crosslinkers, initiators, and porogens because the templates have similar characteristics [73]. Examples of templates that have been used for MT-MIPs are cephalosporin antibiotics (cefoperazone, cefazolin, and cephalexin), non-steroidal anti-inflammatory drugs (NSAIDs; naproxen, ibuprofen, and diclofenac), and sulphonamide antibacterial drugs (sulphanilamide, sulphacetamide, sulphadiazine, sulphathiazole, sulphamerazine, sulphamethizole) [32,33,73,75,76,77].

#### 2.3.2. Functional Monomer

The characteristics of functional monomers affect the success of creating MIP. Functional monomers are an important factor for binding interactions in molecular imprinting technology. The strength and type of template–monomer interactions are taken into consideration while choosing the optimal monomers for the synthesis of imprinted materials to create a particular donor–receptor interaction because the success of molecular recognition depends on the creation of a stable template–monomer complex [77,78]. To maximise their interactions and to create highly specialised holes created for the template molecule, it is imperative to match the activity of the functional monomer to that of the imprinted molecule (template). In addition, the choice of monomer must consider its interactions with the solvent: the monomer must be stable and soluble in the chosen solvent [79]. The best monomers can be chosen that will likely exhibit cage effects or strong interactions in the specified solvent. This will boost the capacity of binding cavities and have an effect on their homogeneity. The monomer also needs to be adapted to a certain polymerisation method [80]. Moreover, the monomers used for MIPs must withstand thermal and chemical changes and are not easily affected by environmental influences [81,82]. The most commonly used monomers are MAA, 2-VP, 4-VP, 1-vinylimidazole, AM, trifluoromethyl acrylic acid (TFMAA), 2-acrylamido-2-methylpropane sulphonic acid, and 2-hydroxyethylmetacrylate (HEMA) [77].

#### 2.3.3. Crosslinker

The crosslinker has the same function in ST-MIP and MT-MIP synthesis, namely binding the functional monomer complex and the template to form a rigid structure [29]. If the MIP formed has a rigid structure, the monomer structure around the template will not change even if the template is removed. Therefore, the use of a crosslinker greatly affects the stability of the MIP that is formed [77]. The amount of crosslinker usage should be carefully considered. Insufficient use of crosslinkers can lead to poor MIP stability and template leakage during synthesis [83]. Meanwhile, the use of too many crosslinkers results in impaired cavity banding between functional monomers and templates so that the sensitivity of the binding site is reduced [29,84].

In selecting the crosslinker used, there are several things that must be considered, namely the functional monomer, the type of bond between the functional monomer and templates, the type of polymerisation, and the solvent [6,84,85,86]. The types of functional monomers and crosslinkers must have the same reactivity so that when random copolymerisation is carried out, the functional groups of monomers can be evenly distributed [6,85]. The bond between functional monomers and templates is also a matter that must be considered because the improper selection of crosslinkers can disrupt the bonds that are formed [84,86]. The type of polymerisation is also a consideration because the presence or absence of free radicals in the polymerisation process will affect the MIP formed [6,85]. The crosslinkers commonly used for free radical polymerisation are EGDMA, divinylbenzene (DVB), and trimethylolpropane-trimethacrylate (TRIM) for non-covalent bonds, and triallyl isocyanurate (TAIC), dicumyl peroxide (DCP), and bis-(1-*tert*-butylperoxy)-1-methylethyl)-benzene (BIPB) for covalent bonds [6,85,87]. In addition, the solvent must also be considered: the crosslinker used must be insoluble in the solvent used. Commonly used crosslinkers for inorganic solvents are *N*,*N*-methylenebisacrylamide and the usual crosslinkers for organic solvents are DVB and EGDMA [6,85].

#### 2.3.4. Porogen

In the MIP process, the nature and volume of the solvent play important roles. The porogen influences how the target and the monomer interact. During polymerisation, the porogen acts as a dispersion medium and helps to form a homogeneous cavity. For MIP synthesis, toluene, chloroform, dichloromethane, or acetonitrile are the most often utilised solvents. In the polymerisation process, the solvent serves to combine all of the constituents (monomer, template, initiator, and crosslinker) into one phase and is what provides macroporous polymers with their pores. To ensure that the resultant MIP has good flow-through properties, the solvent should produce large pores; as the volume of the solvents increases, so do the polymer′s pore sizes. For this reason, the term porogen is frequently used to describe the solvent [77,82].

The interaction between the template molecule and the functional monomer can be impacted by the porogen′s polarity. Organic solvents that tend to be non-polar, with low dielectric constants, such as toluene, acetonitrile, and chloroform, are often used for polar non-covalent printing because they enhance the formation of complexes that facilitate polar non-covalent interactions such as hydrogen bonding. The interaction force between the analyte and the polymer tends to decrease when a more polar solvent is used because more polar solvents tend to detach the non-covalent interactions in the prepolymer complex, especially protic solvents which leads to a high degree of hydrogen bond disruption in the monomer template [77,86,88,89].

Mansour et al. [90] listed the requirements that must be fulfilled before a substance may be utilised as a porogen. (1) The porogen must be inert, have a high boiling point, and not polymerise or react with monomers, other materials, or other substances in the polymerisation mixture. (2) If a thermal initiator is to be used, a solvent with a boiling point higher than the initiator′s decomposition temperature is typically chosen. The porogen must also be compatible with the initiation method. (3) When combined with monomers and crosslinkers, porogens must be miscible with one another and capable of forming homogenous solutions. The partition coefficient (log P), polarity index (PI), and solubility factors are also additional selection criteria for pyrogens. MIPs with small diameters and substantial surface areas can be made by solvents with strong dipole moments, and low log P. MIPs with small pores can also form when solvents with a high polarity index are used. The Hildebrand solubility parameter (δ) of the solvent can be used in selecting the combination of porogens by comparing the value of δ in each solvent with the value of δ in the monomer. In general, organic solvents are considered good if the difference in the value of δ < 1.7 MPa12 [90].

#### 2.3.5. Initiator

The most widely used polymerisation method for the synthesis of ST-MIPs and MT-MIPs is free radical polymerisation. This process consists of three stages, namely initiation, propagation, and termination [91,92]. An initiator compound is required during the initiation stage. As the name implies, the initiator serves to start polymerisation [93]. Initiation occurs when the initiator breaks down into fixed original molecule pieces called starter pieces. These pieces contain free electrons that find a partner by bonding to one of the carbon double bonds in the functional monomer and start the polymerisation process. Therefore, the use of an initiator is very important because it can determine the success of the polymerisation process [92]. A good initiator is a compound that can be decomposed easily. Compounds that are commonly used as initiators are peroxy compounds and azobis. 2,2′-Azo-isobuttyronitrile (AIBN) is used as the initiator because it can be decomposed by photolysis and thermolysis (50–70 °C) [28,29].

## 3. A Review of MT-MIPs That Have Been Created

The first study to synthesise an MT-MIP used salicylic acid and hydrocortisone as the templates [94]. In this 1998 study, the authors used HEMA as the functional monomer and EGDMA as a crosslinker. They produced MIPs in acetonitrile solutions of salicylic acid, hydrocortisone, and their mixture. The MT-MIPs had a higher absorption capacity than the control, which was higher than the control for aspirin extraction. MT-MIPs have mostly been applied as absorbents in solid phase extraction (SPE) (Tables 3–5). SPE itself has been applied in the field of extraction and separation for a long time [95,96]. However, general absorbents cannot absorb the analyte optimally due to the presence of a complex matrix mixed with the analyte [97,98]. Therefore, we need absorbents that have high selectivity and are stable, reusable, low cost, and time efficient [98,99,100]. As previously explained, MT-MIP has these advantages. That is why the use of MIP as an absorbent in SPE has good potential. In some studies, the application of MT-MIP is not specific [54,67,101,102]. However, MT-MIPs can be used in analytical processes involving sorbents and analytes in the purification or separation processes.

### 3.1. Template

#### 3.1.1. Drugs

The most widely used templates in MT-MIP synthesis are drugs because some drugs leave residues that are toxic to the environment and the body. Hence, there is an urgency to analyse drug levels in the environment and humans [103,104,105,106,107]. The existence of drug residues in this environment is due to industrial waste, expired drug products, hospital waste, and the metabolic products of human and animal excretion [105]. Therefore, an efficient, low-cost, highly sensitive, and fast analytical method is required to detect these drugs [108,109,110]. MT-MIPs represent a solution to this problem.

The drugs used as templates in MT-MIP are mostly drugs in the same class (see Table 3). This is to facilitate the selection of other components, such as monomers, crosslinkers, initiators, and porogens [28]. The more similar the basic structure of a compound, the more similar the physicochemical properties of the compound will be. The physicochemical properties of the template determine the other components that will be selected [69], for example, selecting monomers considering their functional groups and porogens based on the polarity of the template [111]. Examples of drugs that have been used for MT-MIP templates are NSAIDs, cephalosporin antibiotics, and sulphonamide antibiotics.

The monomers that have been used in MT-MIP are mostly monomers for non-covalent molecular imprinting procedures, namely MAA, AM, 2-VP, 4-VP, 4-vinylbenzoic acid, and APTES. Likewise, a crosslinker is used to form non-covalent interactions. The most widely used crosslinker is EGDMA. Non-covalent molecular imprinting has been chosen because of the simple method and easy template removal process [117]. The porogens used are mostly non-polar. However, semi-polar porogens have also been chosen based on the polarity of the template used [111]. Some of the porogens that have been used in MT-MIP synthesis are toluene for NSAIDs and sulpha antibiotics and acetonitrile for cephalosporin antibiotics. Free radical polymerisation is mostly used for MT-MIP synthesis [118]. The use of initiators is very important because the success of free radical polymerisation depends on this molecule. The initiator commonly used is azobis, and other initiators include AIBN, 1,1′-azobis-(cyclohexanecarbonitrile) (AHCN), 2,2′-azobis (2,4-dimethyl valeronitrile) (AMVN), and 4,4′-azobis (4-cyanovaleric acid) (ACV) [32,54,62,75,117].

The most widely used polymerisation method in the synthesis of MT-MIPs with drug templates is bulk polymerisation. As previously mentioned, bulk polymerisation is used because the process is simple and universal [35]. Although it requires sieving and grinding, the resulting MT-MIP still has good sensitivity and reusability. This is evidenced by the maximum absorption capacity (Qmax) and IF values of the formed MT-MIP. MT-MIP with cefoperazone, cefazolin, and cephalexin templates, functional MAA monomer, EGDMA crosslinker, AIBN initiator, and ethanol porogen produced Qmax values of 0.0027, 0.0029, and 0.0026, and IF values of 4.1, 3.3, and 3.4 for cefoperazone, cefazolin, and cephalexin, respectively [75]. These values indicate that the formed MT-MIP has good sensitivity and much better performance than non-imprinted polymer (NIP). Other studies have produced good Qmax and IF values using bulk polymerisation (Table 3). In addition to bulk polymerisation, several studies have used precipitation polymerisation [113,115]. Duan et al. [113] synthesised MT-MIP using an NSAID template and obtained IF values of 3.5–76 (Table 3). There is also a study using multi-step swelling polymerisation [54,65]. This method is rarely used because it is difficult to carry out due to the complicated procedure, and it requires many types of reagents and special tools. However, the resulting MT-MIP had uniform size, good selectivity, and good reusability [53]. 

In addition, MT-MIP has excellent reusability and reproducibility. This is evidenced by MT-MIP, which could be used for re-extraction five times for different analyte targets without experiencing significant loss [75]. This shows that MT-MIP has better potential than ST-MIP in terms of the efficiency of the MIP manufacturing process [5]. The synthesis is streamlined, one process for all templates rather than separate processes for each template, and the resulting MT-MIP has good sensitivity, selectivity, and reusability. 

There are not many studies using the same composition and type of template, functional monomer, crosslinker, and porogen between ST-MIP and MT-MIP. Therefore, it is difficult to make a comparison between ST-MIP and MT-MIP due to the lack of study resources. There are several studies using the same template, functional monomer, and crosslinker. However, the composition of the two studies is different, making it hard to compare.

Table 4 shows a comparison of ST-MIPs and MT-MIPs generated using the same monomer and template. ST-MIPs have higher Qmax and IF values than MT-MIPs. In one study, the authors used ibuprofen, naproxen, ketoprofen, diclofenac, and clofibric acid as templates and obtained an IF value of 5.2 for diclofenac [113]. This value is close to the ST-MIP IF, which is 7.185 [119]. However, Nkosi et al. [116] obtained an IF that was quite far from the ST-MIP IF, with a difference of 5.99. There have also been marked differences in ST-MIPs and MT-MIPs generated using fenoprofen and cephalexin templates (Table 4). This marked difference is influenced by many things, such as the polymerisation method and the template:monomer:crosslinker ratios [4]. In MT-MIP, the binding cavity formed is specific for each template used. That is, the binding cavity in an ST-MIP is only for one template, while in an MT-MIP, the cavity is divided into several templates. For example, one study synthesised ST-MIP using cephalexin as a template, MAA as functional monomer, and EGDMA as crosslinker with a ratio of 1.01:4.03:20.1 mmol (template:monomer:crosslinker), resulting in an IF of 14.7 [120]. Another study synthesised MT-MIP using cephalexin as one of the templates, MAA as a functional monomer, and EGDMA as crosslinker with a ratio of 0.127:0.557:12,713 mmol (template:monomer:crosslinker). In the 0.127 mmol template, the cephalexin composition is 0.046 mmol. The formed MT-MIP has an IF value of 3.4 against cephalexin [75]. The difference in the ratio of the number of cephalexin templates used is the cause of IF deficiency in MT-MIP. In addition, the two studies used significantly different amounts of porogens. The ST-MIP used 5.6 mL acetonitrile and MT-MIP used 60 mL acetonitrile [75,120]. Therefore, the Qmax of an MT-MIP is smaller than that of an ST-MIP [75,113,116,119,120,121]. Although MT-MIP has lower Qmax and IF values than ST-MIP (Table 4), it does not make MT-MIP worse than ST-MIP. That is because the main purpose of developing MT-MIP is to isolate several different compounds simultaneously or continuously without having to synthesise new MIP. The IF of MT-MIPs is still maintained at >1 (Table 3 and Table 4). This shows that the formed MT-MIPs have better sensitivity than the NIPs and show great potential [4]. 

#### 3.1.2. Organic Compounds

Organic compounds, including phenolic compounds, are often used as templates [9]. Phenolic compounds, particularly chlorinated compounds, are harmful and persistent in the environment at low concentrations and have been added to the priority pollutant list [9,122]. Hence, researchers have synthesised MT-MIPs to recognise these compounds, including polycyclic aromatic hydrocarbons (PAHs) as templates. PAHs are a type of persistent organic pollutant that is typically detected in low concentrations in the environment. Household human and chemical waste, automobile exhaust products, storm water run-off from both impervious and pervious areas such as roads, parking lots, and construction sites, industrial effluents from chemical manufacturing, and carbonaceous waste incineration are all sources of PAHs in urban areas. They subsequently make their way into wastewater treatment plants through the sewage system [61,123,124,125,126]. PAHs have also been added to priority pollution lists due to environmental and possibly carcinogenic health concerns [126,127].

Ma et al. [102] used nitrogen compounds (aniline, indole, and 3-methylindole) in oil as templates; these compounds are pollutants and harmful to the environment. Other research has also used organic compounds that are harmful to the environment as templates, including dibutyl phthalate (DBP), diethyl phthalate, and dimethyl phthalate, which are phthalate esters (PAEs), synthetic organic compounds intensively used as important additives in plastic industry. PAEs can escape from plastic materials into the environment because they are not chemically linked to the polymeric matrix [101]. In one study, the authors used methyl parathion and quinalphos, organophosphorus pesticides (OPPs) that are irreversible acetylcholine esterase (AChE) inhibitors, as templates. OPPs are frequently used to control pests in agriculture. However, OPP residues are a human health risk because prolonged exposure to OPPs might damage several organs [128].

For the enrichment of tracing this toxic organic compound, many traditional and novel pretreatment strategies have been reported, including SPE. It is a simple and easy-to-automate approach that has been widely used in environmental sectors. Target analytes are frequently retained on their functionalised surface by physicochemical interaction for the most commonly used SPE sorbents. Other matrix molecules may unintentionally be retained on SPE sorbents in addition to the target. Thus, MIPs with artificial recognition cavities complementary to the template molecules in shape, size, and chemical functionality can specifically rebind the template molecules from the complicated matrices that are being utilised for SPE [9,27,129].

In addition to analysing components that are harmful to the environment and health, MIPs with organic compounds as a template are used to separate compounds from herbal plants. Researchers have used phenyl pyruvic acid and DL-tyrosine as templates. These compounds contain an amino (-NH2) group, a carboxylic acid (-COOH) group, and a keto acid (-COCOOH) group, which is present in dencichine. This compound is the primary component of *Panax notoginseng* and other traditional Chinese medications; it increases platelets and promotes haemostasis [130,131]. Numerous analytical techniques have been developed for its identification due to the positive and negative pharmacological activities of dencichine. Even though extraction and purification of individual bioactive components from traditional Chinese medicines and natural products are tough, they is necessary for drug development and pharmaceutical analysis. SPE with MIPs could be particularly useful to extract compounds due to high selectivity, mechanical/chemical stability, and inexpensive preparation. Indeed, this technique has been used to selectively extract dencichine from *P. notoginseng* [132].

As shown in Table 5, the most widely used functional monomer when using organic compounds as templates is MAA. It is preferred because it interacts with neutral or basic target molecules. In fact, MAA can develop hydrogen and ionic bonds with basic molecules and operate as an H^+^ donor and acceptor with strong dipole–dipole interactions [79]. AM and 4-VP have also been used as functional monomers. The most widely used crosslinker is EGDMA, which can increase resistance to heat, solvents, and abrasion in the copolymerisation process [133]. In addition, EGDMA can form polymers with good flexibility; EGDMA creates a space between the polymer chains that facilitates analyte molecule accessibility and attachment to the functional groups of EGDMA. The hydrophilicity and polarity of EGDMA provide it with a high affinity for the aqueous phase, and it does not exhibit steric constraints when used as an adsorbent [134].

MT-MIPs synthesised with organic molecules as templates have presented good results, with IF values >1 (Table 5). Using the seeded emulsion polymerisation for aniline, indole, and 3-methylindole as templates produced an IF of 5.5, 4.15, and 3.68. Using two-stage precipitation polymerisation for aniline, indole, and 3-methylindole produced an IF of 4.89, 2.89, and 2.83 [102]. In another study, the IF for 1-naphthol, 9-phenanthrole, and 9-hydroxyfluorene was 3.7, 3.1, and 2.6 [61].

Wang et al. [139] synthesised MT-MIP to synthesise 17 triazine herbicides. MT-MIP was synthesised with atrazine and prometryn as a template, MAA functional monomer, TRIM crosslinker, AIBN initiator, and acetonitrile as porogen. In this study, the synthesis of NIP and ST-MIP for each template was also carried out. MT-MIP produces a selectivity percentage of 72.9%, while NIP produces a selectivity percentage of 30.9%. This shows that the formed MT-MIP has good selectivity. In addition, in comparison with ST-MIP, MT-MIP is more effective for the extraction of several analytes at one time. This is evidenced by the inability of ST-MIP to absorb chloro-triazine while MT-MIP can. The formed MT-MIP also has good reusability. After being used 25 times for five cycles, it was observed that the extraction efficiency was still in the range of 77.8–112.1%. Even after 25 cycles, MT-MIP did not suffer any significant damage [139].

MT-MIPs generated using organic compound templates produced good Qmax values (Table 5), indicating their good selectivity. In one study, MT-MIP was synthesised using MAA as a monomer and methyl parathion as one of the templates; the Qmax was 13.13 [128]. In another study, methyl parathion was used to generate ST-MIP with the same monomer, and the Qmax was 12.9 [140]. However, IF cannot be compared between the study because one of the studies did not include this measurment [128]. In addition, ST-MIP and MT-MIP cannot be compared. This is because the two studies have different variables, as explained in the drugs section.

#### 3.1.3. Proteins

Researchers have also used proteins as templates, namely 17ß-oestradiol, oestriol, and diethylstilbestrol [66]. The latter is an endocrine-disrupting compound (EDC) that can interfere with the regulatory function of wildlife and humans by mimicking or antagonising endogenous hormones [141,142,143]. EDCs can accumulate in the environment and food chains. This is due to pollution from cosmetic products, contraceptives, hormone replacement therapy, and rapid human population growth [144,145,146]. Manifestations of these effects include a decrease in the quality and quantity of human sperm, an increased risk of prostate and breast cancer, and feminisation in marine life [147,148,149,150]. Another study also synthesised MT-MIP using protein templates in the form of bovine haemoglobin and bovine serum albumin [67].

Both studies were carried out using solvothermal polymerisation with APTES and PTMOS as functional monomers and produced good Qmax and IF values (Table 6). In addition, the formed MT-MIPs had good reusability. After six absorption cycles, Qmax remained >90% of the initially formed MT-MIP value [66]. This indicates that the use of multiple templates in MT-MIP does not make the MIP formed less sensitive or selective. This also makes MT-MIPs superior to ST-MIPs because they do not require separate MIP synthesis for each template.

## 4. Conclusions and Future Aspects

MT-MIPs have great potential in chemical synthesis and analysis. MT-MIPs show good sensitivity, selectivity, and reusability compared to NIP. Furthermore, many templates, functional monomers, and crosslinkers can be formulated as MT-MIPs and have a high success rate. This is evidenced by the good Qmax, IF (maintained > 1), and reusability. Despite this, in some studies, MT-MIP has lower Qmax and IF values than ST-MIP, but it does not make MT-MIP worse than ST-MIP. That is because the main purpose of developing MT-MIP is to isolate several different compounds simultaneously or continuously without having to synthesise new MIP, so it is more cost-effective, has a short analysis time, and is easy to prepare. Despite their benefits, a lot of research is required to ensure MT-MIPs can be utilised as greener and more efficient options compared with ST-MIPs. Moreover, research comparing ST-MIP and MT-MIP with the same template from different chemical compounds is still required to elucidate the benefits of MT-MIPs. 

## Figures and Tables

**Table 1:** Single templates used for molecularly imprinted polymers (MIPs).

Template Type	Template	Polymerisation Method	Application	Reference
Drugs	Valsartan	Bulk polymerisation	SPE	[12]
	Ofloxacin	Surface-initiated atom transfer radical polymerisation	SPE	[13]
	Dexamethasone	Surface imprinting polymerisation	Not mentioned, but it can be utilised in any application where a sorbent is required to purify and concentrate a target analyte	[14]
	Olanzapine	Co-precipitation polymerisation	Medical (brain drug delivery)	[15]
	Pseudoephedrine	Free radical polymerisation	SPE	[16]
	Pentamidine	Precipitation polymerisation	SPE	[17]
	Ibuprofen	Precipitation and bulk polymerisation	SPE	[18]
Organic compounds	Melamine	Precipitation polymerisation	SPE	[19]
	Dibutyl phthalate	Bulk polymerisation	GC-MS	[20]
	Auramine O	Surface imprinting polymerisation	Not mentioned, but it can be utilised in any application where a sorbent is required to purify and concentrate a target analyte	[21]
	Catechins	Bulk polymerisation	SPE	[22]
	Genistein	Electro polymerisation	LFD	[23]
Proteins	Bovine haemoglobin	Surface imprinting polymerisation	Not mentioned, but it can be utilised in any application where a sorbent is required to purify and concentrate a target analyte	[24]
	17β-oestradiol	One-pot surface imprinting method	HPLC	[9]
	17β-oestradiol	Free-radical thermal-polymerisation	SPE	[25]

GC-MS: gas chromatography–mass spectrometry; HPLC: high-performance liquid chromatography; LFD: lateral flow devices; SPE: solid phase extraction.

**Table 2:** Summary of polymerisation methods for multi-template molecularly imprinted polymers.

Polymerisation Method	Procedure	Advantages	Disadvantages	Reference
Bulk polymerisation	Polymerisation is carried out using an organic solvent, followed by sieving and grinding	- Universal - Easy and simple method	- Shape, particle size, and active sites tend to be heterogeneous - The binding cavity can be damaged in the grinding process	[34,35,36,37,38]
Precipitation polymerisation	Polymerisation occurs in solution; precipitation occurs after the polymer is formed and makes it insoluble in solution	- One step preparation - Same shape and particle size and high yields	- Requires large amounts of templates - Requires more solvent than bulk polymerisation	[37,39,40,41]
Emulsion polymerisation	Polymerisation requires surfactants to emulsify the organic phase and the aqueous phase	- The product is monodisperse - Very small size, around 10–100 nm	- Requires a surfactant - The process of removing surfactants is difficult - Surfactants can affect the efficiency of polymerisation because it affects the interaction between templates and monomers	[42,43]
Suspension polymerisation	Polymerisation is carried out using water as a medium	- One step preparation - The particle shape is the same and round - Can be made on a large scale	- The large particles are micromillimetres - The use of water tends to be incompatible with the procedure	[44,45,46,47,48]
Surface polymerisation	Surface grafting of thin molecularly imprinted polymer layers is used in polymerisation	- The product is monodisperse - Thin imprinted surface	- A difficult and complicated process - Takes a long time	[21,49,50,51,52]
Multi-step swelling/seed polymerisation	The initiator′s oil-in-water emulsion is used to generate spherical particles, which then swell	- The shape and size of the particles are the same	- A complicated process - Takes a long time	[53,54,55]
Monolithic imprinted polymerisation	A mixture of porogens is used to dissolve the template, functional monomer, crosslinker, and initiator, which is then degassed and put into a stainless-steel column	- Simple method - One step preparation - Free radical polymerisation	- Extensive optimisation is necessary for each new template system	[56,57]

**Table 3:** Drugs used as templates in multi-template molecularly imprinted polymers.

Template	Monomer	Polymerisation Method	Qmax (mg/g)	IF	Application of MT-MIP	Reference
Acetaminophen Codeine	APTES	Bulk polymerisation	8.76 6.29	4.09 4.16	SPE	[59]
Cefoperazone Cefazolin Cephalexin	MAA	Bulk polymerisation	0.0027 0.0029 0.0026	4.1 3.3 3.4	SPE	[75]
Ginsenoside Rb1 Ginsenoside Rg1 Notoginsenoside R1	AM	Surface polymerisation	-	2.11	SPE	[112]
Ibuprofen Naproxen Ketoprofen Diclofenac Clofibric acid	2-VP	Precipitation polymerisation	-	7.6 4.8 3.5 5.2 4.1	SPE	[113]
Naproxen Ibuprofen Diclofenac	2-VP	Bulk polymerisation	-	-	SPE	[73]
Naproxen Ibuprofen Diclofenac	2-VP	Bulk polymerisation	-	-	SPE	[33]
Naproxen Ibuprofen Diclofenac	2-VP	Bulk polymerisation	-	-	SPE	[32]
Naproxen Ibuprofen Diclofenac	2-VP	Bulk polymerisation	-	-	SPE	[74]
Rutin Scoparone Quercetin	γ-Aminopropyltriethoxysilane-methacrylic	Bulk polymerisation	0.025 0.057 0.025	-	SPE	[114]
Sulphadiazine Sulphathiazole Sulphamerazine Sulphamethazine Sulphamethoxazole Sulphadoxine	4-vinylbenzoic acid	Precipitation polymerisation	-	-	SPE	[115]
Sulphanilamide Sulphacetamide Sulphadiazine Sulphathiazole Sulphamerazine Sulphamethizole	APTES	Bulk polymerisation	6 5.1 4.2 9.8 3.7 9.8	1.56 2.7 1.7 3.9 1.13 4.52	SPE	[76]
Chlorpromazine Bromopromazine	MAA	Multi-step swelling polymerisation	-	-	Not mentioned, but it can be utilised in any application where a sorbent is required to purify and concentrate a target analyte	[54]
Genistein Naringin	4-VP	Multi-step swelling polymerisation	-	-	SPE	[65]
Ibuprofen Naproxen Diclofenac Fenoprofen Gemfibrozil	2-VP	Bulk polymerisation	3.388 4.852 5.643 3.655 4.897	1.113 1.275 1.195 1.174 1.159	SPE	[116]
Thiamethoxam Thiacloprid	2-VP	Precipitation polymerisation	-	-	SPE	[62]

AM: acrylamide; APTES: 3-aminopropyltriethoxysilane; IF; imprinting factor; MAA: methacrylic acid; Qmax: maximum adsorption capacity; SPE: solid phase extraction; VP: vinyl pyridine.

**Table 4:** Comparison of single-template and multi-template molecularly imprinted polymers generated using drug templates.

Monomer	Template	Qmax (mg/g)		IF		Reference
		ST-MIP	MT-MIP	ST-MIP	MT-MIP	
2-VP	Diclofenac	324.8	5.643	7.185	1.195	[113,116,119]
			-		5.2	
2-VP	Fenoprofen	38.8	3.655	1.9	1.174	[116,121]
MAA	Cephalexin	-	0.0026	14.7	3.4	[75,120]

2-VP: 2-vinylpyridine; 4-VP: 4-vinylpyridine; IF; imprinting factor; MAA: methacrylic acid; MT-MIP, multi-template molecular imprinted polymer; Qmax: maximum adsorption capacity; ST-MIP, single-template molecular imprinted polymer; VP: vinyl pyridine.

**Table 5:** Organic molecules used as templates for multi-template molecularly imprinted polymers.

Template	Monomer	Polymerisation Method	Qmax (mg/g)	IF	Application of MT-MIP	Reference
PAHs	P-vinylbenzene	Not mentioned	-	-	SPE	[126]
Aniline Indole 3-methylindole	MAA, AM, and 4-VP	Seeded emulsion polymerisation	47.49 42.45 39.87	5.5 4.15 3.68	Not mentioned, but it can be utilised in any application where a sorbent is required to purify and concentrate a target analyte	[102]
Aniline Indole 3-methylindole	MAA, AM, and 4-VP.	Two-stage precipitation polymerisation	46.39 29.34 25.81	4.89 2.89 2.83	Not mentioned, but it can be utilised in any application where a sorbent is required to purify and concentrate a target analyte	[102]
Capsaicin Dihydrocapsaicin Eugenol	MAA and AM	Bulk polymerisation	0.04625 0.04538 0.04738	-	Not mentioned, but it can be utilised in any application where a sorbent is required to purify and concentrate a target analyte.	[135]
Dimethyl phthalate Diethyl phthalate Dibutyl phthalate	MAA	Multi-step polymerisation	0.95 1.38 7.09	-	Not mentioned, but it can be utilised in any application where a sorbent is required to purify and concentrate a target analyte.	[101]
Phenol 4-chlorophenol 2,4,6-trichlorophenol 2,4-dichlorophenol 2-chlorophenol 2,6-dichlorophenol	MAA	Precipitation polymerisation	-	-	SPE	[9]
1-naphthol 9-phenanthrol 9-hydroxyfluorene	MAA	Precipitation polymerisation	-	3.7 3.1 2.6	SPE	[61]
2,4,6-triaminopyrimidine 4-hydroxy-2-butanone Imidazole	MAA	Bulk polymerisation	-	-	SPE	[136]
Methyl parathion Quinalphos	MAA	Bulk polymerisation	13.13 31.47	-	SPE	[128]
Chlorogenic acid Rutinum	AM	Bulk polymerisation	-	-	SPE	[137]
Phenyl pyruvic acid DL-tyrosine	4-VP	Bulk polymerisation	-	-	SPE	[132]
*N*-*tert*-butyloxycarbonyl-l-phenylalanine *N*-acetyll- phenylalaninyl-l-tryptophanyl methyl ester yohimbine	MAA	Bulk polymerisation	-	-	Not mentioned, but it can be utilised in any application where a sorbent is required to purify and concentrate a target analyte.	[138]
*AtrazinePrometryn*	MAA	Bulk Polymerisation	-	-	SPE	(wang)

4-VP: 4-vinylpyridine; AM: acrylamide; IF: imprinting factor; MAA: methacrylic acid; MT-MIP, multi-template molecularly imprinted polymer; Qmax: maximum adsorption capacity; SPE: solid phase extraction.

**Table 6:** Proteins used as templates in the synthesis of multi-template molecularly imprinted polymers.

Template	Monomer	Polymerisation Method	Qmax (mg/g)	IF	Application of MT-MIP	Reference
Bovine haemoglobin Bovine serum albumin	APTES and OTMS	Solvothermal polymerisation	73.12 44.25	5.54 4.98	Not mentioned, but it can be utilised in any application where a sorbent is required to purify and concentrate a target analyte	[67]
17ß-oestradiol Oestriol Diethylstilbestrol	APTES and PTMOS	Solvothermal polymerisation	3.74 6.02 6.89	3.2 6.38 5.69	SPE	[66]

APTES: 3-aminopropyltriethoxysilane; OTMS: octyltrimethoxysilane; PTMOS: phenyltrimethoxysilane; Qmax: maximum adsorption capacity; SPE: solid phase extraction.

## Data Availability

Not applicable.
